# SRF Is Required for Maintenance of Astrocytes in Non-Reactive State in the Mammalian Brain

**DOI:** 10.1523/ENEURO.0447-19.2020

**Published:** 2021-01-28

**Authors:** Monika Jain, Soumen Das, Paul P. Y. Lu, Garima Virmani, Sumitha Soman, Surya Chandra Rao Thumu, David H. Gutmann, Narendrakumar Ramanan

**Affiliations:** 1 Centre for Neuroscience, Indian Institute of Science, Bangalore, Karnataka 560012, India; 2 Jiangsu Hengrui Medicine, Cambridge, MA 02139; 3 Department of Neurology, Washington University School of Medicine, St. Louis, MO 63110

**Keywords:** astrogliosis, gliosis, reactive astrocytes, serum response factor

## Abstract

Astrocytes play several critical roles in the normal functioning of the mammalian brain, including ion homeostasis, synapse formation, and synaptic plasticity. Following injury and infection or in the setting of neurodegeneration, astrocytes become hypertrophic and reactive, a process termed astrogliosis. Although acute reactive gliosis is beneficial in limiting further tissue damage, chronic gliosis becomes detrimental for neuronal recovery and regeneration. Several extracellular factors have been identified that generate reactive astrocytes; however, very little is known about the cell-autonomous transcriptional mechanisms that regulate the maintenance of astrocytes in the normal non-reactive state. Here, we show that conditional deletion of the stimulus-dependent transcription factor, serum response factor (SRF) in astrocytes (*Srf*^GFAP^CKO) results in astrogliosis marked by hypertrophic morphology and increased expression of GFAP, vimentin, and nestin. These reactive astrocytes were not restricted to any specific brain region and were seen in both white and gray matter in the entire brain. This astrogliosis persisted throughout adulthood concomitant with microglial activation. Importantly, the *Srf* mutant mouse brain did not exhibit any cell death or blood brain barrier (BBB) deficits suggesting that apoptosis and leaky BBB are not the causes for the reactive phenotype. The mutant astrocytes expressed more A2 reactive astrocyte marker genes and the *Srf*^GFAP^CKO mice exhibited normal neuronal numbers indicating that SRF-deficient gliosis astrocytes are not neurotoxic. Together, our findings suggest that SRF plays a critical role in astrocytes to maintain them in a non-reactive state.

## Significance Statement

In response to CNS injury, infection and in neurodegeneration, astrocytes undergo complex structural and physiological changes termed as reactive gliosis. Currently, the molecular mechanisms that regulate the non-reactive state of the astrocytes are poorly understood. We report that the serum response factor (SRF) transcription factor is required for the maintenance of astrocytes in the non-reactive state such that its conditional deletion in astrocytes results in widespread reactive astrogliosis. The SRF-deficient reactive astrocytes are persistent, non-proliferating and are not caused by cell death or impaired blood brain barrier (BBB) integrity. In this regard, SRF regulates reactive astrocyte generation in the mammalian brain in a cell-autonomous manner.

## Introduction

As an essential part of the CNS, astrocytes play critical roles in nearly every facet of its development and function including ion and neurotransmitter homeostasis, maintenance of the blood brain barrier (BBB), synapse formation and elimination, and synaptic transmission (Barres, 2008; Kimelberg, 2010; Kimelberg and Nedergaard, 2010). In addition, astrocytic dysfunction are central in several CNS disorders such as epilepsy, amyotrophic lateral sclerosis and Alzheimer’s disease (Seifert et al., 2006; Phatnani and Maniatis, 2015). In response to CNS injuries and pathologies, astrocytes undergo a spectrum of gene expression as well as physiological and structural changes, a process known as reactive astrogliosis (Burda and Sofroniew, 2014; Liddelow and Barres, 2017). These astrocytic responses depend on the severity of the CNS trauma and can range from transient responses lasting a few days to a more permanent glial scar formation (Sofroniew, 2009, 2015).

Reactive astrogliosis is largely considered beneficial to the CNS, where reactive astrocytes provide protection by several mechanisms, ranging from efficient uptake of excitotoxic glutamate, preventing oxidative stress and reducing edema, to restricting inflammation, facilitating BBB repair and restricting spread of infection (Escartin and Bonvento, 2008; Hamby and Sofroniew, 2010; Pekny and Pekna, 2014). However, astrogliosis can also cause detrimental effects wherein reactive astrocytes inhibit CNS regenerative responses, contribute to neuroinflammation, generate reactive oxygen species and cause cell death (Pekny and Pekna, 2014; Sofroniew, 2014). Previous studies have identified several extracellular factors and intracellular signaling pathways that regulate different aspects of astrogliosis including cytokines, growth factors, purines, endothelin-1, BMP receptors, Eph4, and Aquaporin 4 (Correa-Cerro and Mandell, 2007; Kang and Hébert, 2011; Sofroniew, 2014).

Currently, we know little about the identities of transcription factors that are necessary to maintain astrocytes in a non-reactive state. Serum response factor (SRF) is a stimulus-dependent transcription factor important for several aspects of nervous system development (Knöll and Nordheim, 2009). SRF has been shown to play a critical role in oligodendrocyte and astrocyte development (Stritt et al., 2009; Lu and Ramanan, 2012) but its functions in astrocytes remain unknown. In this study, we conditionally ablated SRF in astrocytes using a GFAP-Cre transgenic line (Bajenaru et al., 2002). The brains of *Srf*^GFAP^CKO mice exhibited reactive astrogliosis starting three weeks of age. These reactive astrocytes were not restricted to any specific region and were seen throughout the brain. The reactive gliosis persisted throughout adulthood with concomitant microglial activation. We did not observe any changes in cell death or BBB integrity, indicating that these extrinsic factors are unlikely the cause of gliosis. Together, our findings suggest that SRF is a critical cell-autonomous regulator of non-reactive state of astrocytes throughout the brain.

## Materials and Methods

### Animals

The *Srf*-floxed mice were previously described (Ramanan et al., 2005) These mice were bred with hGFAP-Cre (generously provided by David Gutmann, Washington University School of Medicine, St. Louis, MO) to obtain *Srf*^f/f^-GFAPCre+/− (*Srf*^GFAP^CKO). *Srf*^f/f^ mice served as control in all experiments. Both male and female mice were used in all the experiments. All experiments were conducted in accordance with the animal care standards and use and approved by the Institutional Animal Ethics Committee. Control and mutant mice were housed together, and cage mates were randomly assigned to experimental groups. All experiments were conducted blinded to the genotype of the mice used.

### Immunohistochemistry

Mice were fixed by transcardial perfusion using 4% paraformaldehyde (PFA). The brains were cryoprotected in 30% sucrose, frozen and stored in −80°C until further use. For staining, 30-μm-thick cryosections were incubated in blocking/permeabilization solution containing 0.3% Triton X-100 and 3% goat serum in PBS (pH 7.4) for 1 h followed by overnight incubation in primary antibody. The brain sections were then washed in PBS and incubated in secondary antibody for 1 h. The sections were finally mounted in DAPI-containing mounting medium (Vector Laboratories). For SRF immunostaining, heat-induced epitope retrieval was conducted by incubating the sections in 10 mm sodium citrate, pH 8.9 for 30 min at 95°C. The following primary antibodies were used: anti-GFAP (1:1000; Sigma-Aldrich catalog #G3893, RRID:AB_477010), anti-GFAP (1:1000; Agilent catalog #Z0334, RRID:AB_10013382), anti-Nestin (1:1000; Millipore catalog #MAB5326, RRID:AB_2251134), anti-Vimentin (1:50; DSHB catalog #40E-C, RRID:AB_528504), anti-S100β (1:1000; Sigma-Aldrich catalog #S2644, RRID:AB_477501), anti-S100β (1:500; Synaptic systems catalog #287006, RRID:AB_2713986), anti-Aldh1L1 (1:100; UC Davis/NIH NeuroMab Facility catalog #73-140, RRID:AB_10673447), anti-Iba1 (1:1000; Wako catalog #019-19741, RRID:AB_839504), anti-phosphohistone H3 (phH3; 1:500; Sigma-Aldrich catalog #H0412, RRID:AB_477043), anti-caspase-3 active (1:1500, Millipore catalog #04-439, RRID:AB_673061), anti-SRF (1:200; Santa Cruz Biotechnology catalog #sc-335, RRID:AB_2255249), and anti-NeuN (1:500; Millipore catalog #MAB377, RRID:AB_2298772). Secondary antibodies were as following: Alexa Fluor 488-, 594-, and 647-conjugated anti-rabbit, anti-chicken, and anti-mouse at 1:1000 dilution (Life Technologies). Biotinylated anti-mouse and anti-rabbit secondary antibodies (1:250; Vector Laboratories) were used along with Vectastain ABC Elite, ImmPACT VIP substrate and ImmPACT DAB substrate kits (Vector Labs). All the images were captured using conventional fluorescence microscopy (Eclipse 80i, Nikon), except the images for SRF immunostaining and BBB measurement, which were captured using a confocal microscope (LSM 880, Zeiss).

### RNA isolation and quantitative real-time PCR

Total RNA was isolated from forebrain of three- to five-week-old control and *Srf*^GFAP^CKO mice using the PureLink RNA Mini kit (ThermoFisher Scientific) as per the manufacturer’s protocol; 2 μg of total RNA was used for first-strand cDNA synthesis using the first strand synthesis kit (Invitrogen Inc.). Quantitative RT-PCR was done with 100 ng of cDNA and KAPA SYBR FAST ABI prism kit (catalog #KK4604) using the following program: 95°C or 3 min followed by 39 cycles of 95°C for 5 s, 55°C for 30 s and 72°C for 40 s. The PCR reaction was conducted in QuantStudio 7 Flex Real-Time PCR System (Invitrogen Biosciences). The primers used were: *Srf*, forward (Fwd), 5′-ACCAGTGTCTGCTAGTGTCAGC-3′ and reverse (Rev), 5′-CATGGGGACTAGGGTACATCAT-3′; *Rps29*, Fwd 5′-CCAGCAGCTCTACTGGAGTCA-3′ and Rev, 5′-AGACTAGCATGATCGGTTCCA-3′. *Il1β*, Fwd, 5′-ATCAACAAGCAATTCCTCGATGA-3′ and Rev, 5′-CAGCATTCGCTTCAAGGACATA-3′; TNFa, Fwd, 5′-CCCTCACACTCAGATCATCTTCT-3′ and Rev, 5′-GCTACGACGTGGGCTACAG-3′; *Ccl2*, Fwd, 5′-TTAAAAACCTGGATCGGAACCAA-3′ and Rev, 5′-GCATTAGCTTCAGATTTACGGGT-3′. Expression of *Srf* and other genes were normalized to that of the housekeeping gene, *Rps29*. The primers for A1, A2, and pan-reactive astrocytes were from a previously published study (Liddelow et al., 2017).

### BBB permeability assay

Two assays were used as previously described (Andreone et al., 2017) to measure the integrity of BBB using 6-mon old *Srf*^GFAP^CKO mice. In the first assay, mice were deeply anesthetized with isoflurane and injected with 20 μl of 10-kDa dextran fluorescein (10 mg/ml, Invitrogen; D1820) into the left ventricle of the heart, and allowed to circulate for 5 min. Their brains were collected and postfixed in 4% PFA overnight, frozen and stored at −80°C; 30-μm-thick cryosections were mounted using mounting media supplemented with DAPI (Vector Labs) and analyzed using a confocal microscope (LSM 880, Zeiss). In the second assay, 10 μl of HRP Type II (5 mg/ml) was administered transcardially and allowed to circulate for 5 min. The brains were collected and immersed in 2% glutaraldehyde in 4% PFA in cacodylate buffer (0.1 m, pH 7.3) at room temperature for 1 h. The brains were then shifted to 4°C overnight and sectioned at 100 μm using a Leica vibratome and processed using ImmPACT VIP kit (Vector Labs). For quantification of dextran fluorescein injection, epifluorescence images (63×) of brain sections were analyzed using ImageJ. Brain sections from the same rostro-caudal position were analyzed. At least 12 different regions were taken and the ratio of the fluorescence or color intensity (outside vs inside the vessel) was measured.

### Quantification of fluorescence intensity and cell numbers

For measuring fluorescence intensity, images were scaled for 10× magnification and normalized to the same exposure time. Ten to 12 areas of field [region of interest (ROI), 500 × 500 μm^2^] in the same rostro-caudal axis were drawn per image, and the intensities were measured using ImageJ (Fiji) after subtracting the background fluorescence from both control and knock-out sections. For the hippocampus, cell numbers or fluorescence intensities were measured in the stratum oriens and stratum radiatum. For cell counts, images were taken at 4× magnification. Four ROIs of 1200 × 1200 μm^2^ (1 mm^2^ for hippocampal CA1 and CA3) in the same rostro-caudal axis were drawn per image, and the number of cells per ROI were counted with cell counter plugin using ImageJ (Fiji). For SRF fluorescence intensity, the SRF fluorescence signal within the DAPI area was quantified using ImageJ.

### Quantification of microglial activation

Brain sections from *Srf*^GFAP^CKO mice and control littermates at different ages (three weeks and three and 12 months) were fluorescently immunostained for Iba1. At least five different cortical regions in the same rostro-caudal axis were taken in all the mice to measure fluorescence intensity. This was then compared between the control and the knock-out mice to get the fold difference in fluorescence. ImageJ was used to measure the percentage increase in fluorescence per unit field and this was compared between control and knock-out groups at all the experimental time points as mentioned in statistical analysis. The area of the field was 500 × 500 μm^2^.

### Terminal deoxynucleotidyl transferase-mediated biotinylated UTP nick end labeling (TUNEL) assay

The TUNEL assay was conducted using Click-IT Plus TUNEL assay kit (Invitrogen, ThermoFisher Scientific) according to the manufacturer’s instructions. Briefly, brains fixed in 4% PFA and 30-μm cryosections were permeabilized with proteinase K solution for 15 min and then incubated with TdT reaction mixture for 60 min at 37°C and subsequently with EdUTP for 30 min. The detection was achieved through click reaction between the dUTP bound alkyne group and a picolyl azide fluorescent dye for 30 min. The slides were washed with 3% BSA in PBS for 5 min and rinsed in 1× PBS. The slides were mounted using mounting medium containing DAPI (Vector Labs) and observed using an epifluorescence microscope (Eclipse 80i, Nikon) using appropriate filters and captured using MetaMorph software. Numbers of TUNEL^+^ cells in the CA1, CA3, and DG regions of entire rostral to caudal brain regions were counted using ImageJ software. The area of the field for counting the number of TUNEL^+^ cells was 250 × 250 μm^2^.

### Statistical analyses

Analyses were done using GraphPad Prism 6. The comparisons between two groups were done using unpaired two-tailed Student’s *t* test. All the statistical details for each experiment, including the *n* value, the statistical test used, *p* value, significance of comparisons is mentioned in the figure legends.

## Results

### *Srf* deletion in astrocytes results in reactive astrogliosis

**Figure 1.**
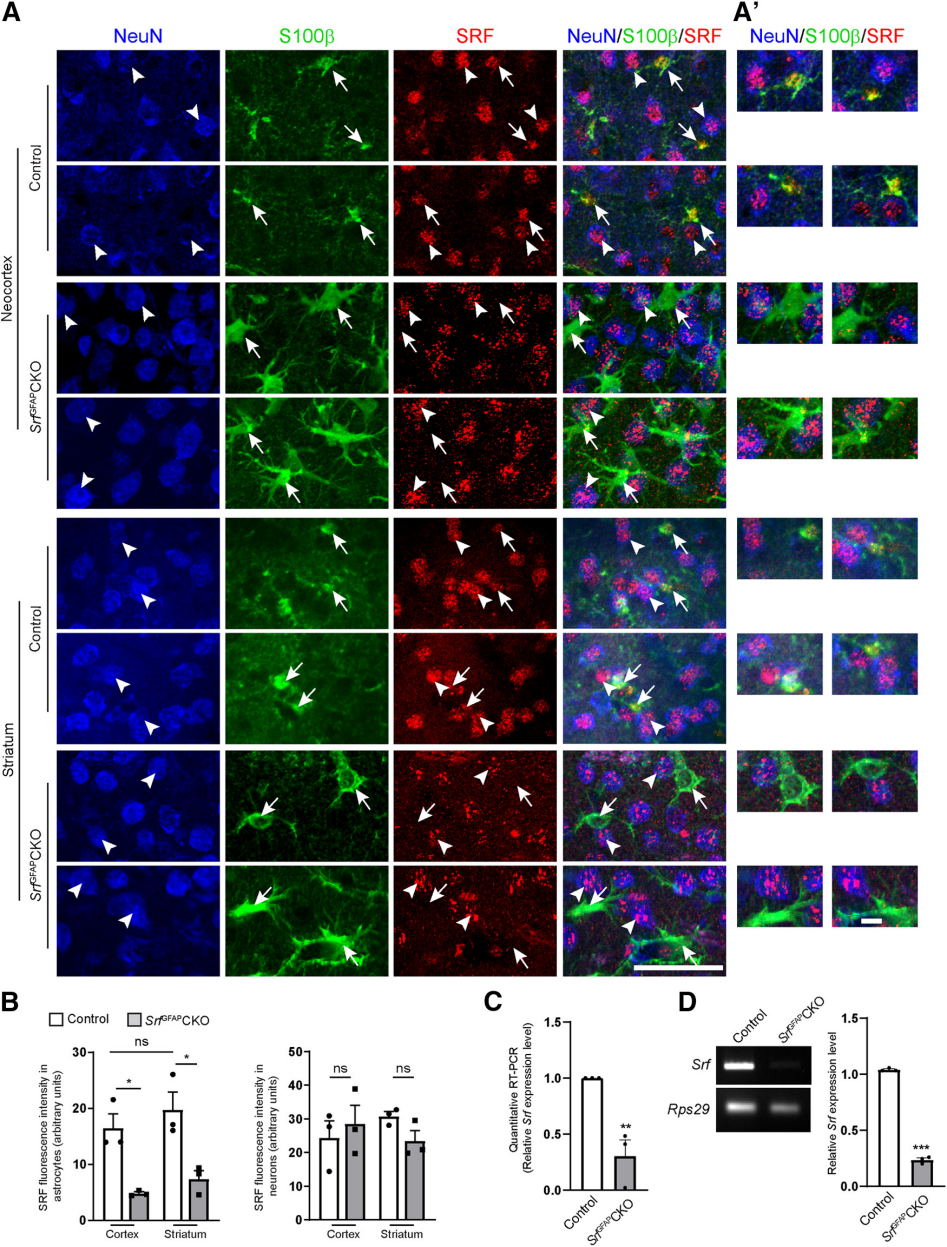
Astrocyte-specific deletion of *Srf* in *Srf*^GFAP^CKO mice. ***A***, Representative images of immunostaining for SRF (red), S100β (green), and NeuN (blue) shows SRF expression in astrocytes (arrows) and neurons (arrowheads) in the cortex and striatum of control mice. SRF expression was seen only in the neurons but not in the astrocytes in *Srf*^GFAP^CKO mice mutant mice. ***A’***, Representative higher magnification images of astrocytes and neurons from ***A*** showing SRF expression in control astrocytes and neurons and absent in mutant astrocytes. ***B***, Quantification of SRF immunofluorescence in S100β^+^ astrocytes from ***A***. At least 15–20 cells per mouse were analyzed (*n* = 3 mice). Astrocytes: cortex, control (16.49 ± 2.52), *Srf*^GFAP^CKO (4.80 ± 0.29); striatum, control (19.72 ± 3.179), *Srf*^GFAP^CKO (7.40 ± 1.47); neurons: cortex, control (24.33 ± 5.05), *Srf*^GFAP^CKO, control (28.47 ± 5.60); striatum, control (30.79 ± 1.40), *Srf*^GFAP^CKO (23.41 ± 3.09). ***C***, Quantitative real-time PCR from whole-brain total RNA shows a significant decrease in *Srf* mRNA expression in the mutant mice relative to control mice; control (1.0 ± 0), *Srf*^GFAP^CKO (0.30 ± 0.14; *n* = 3 mice). ***D***, Semi-quantitative PCR from whole-brain total RNA shows a significant decrease in *Srf* mRNA expression in the mutant mice. *Rps29* expression served as the loading control (*n* = 3 mice). Control (1.04 ± 0.01), *Srf*^GFAP^CKO (0.24 ± 0.02; *n* = 3 mice). Scale bars: 50 μm (***A***) and 20 μm (***A’***); *p < 0.05, **p < 0.005, ***p < 0.0005; ns, not significant. Two tailed *t* test. Data are mean ± SEM.

To study the function of SRF in astrocytes, we generated *Srf*^GFAP^CKO conditional knock-out mice using a hGFAP-Cre transgenic mouse line, in which Cre expression was found to occur predominantly in astroglial progenitor cells starting at embryonic day (E)16.5 (Bajenaru et al., 2002). *Srf*^GFAP^CKO mice were obtained in normal Mendelian ratio, appeared normal at birth, and did not exhibit any gross morphologic deficits such as neocortical lamination and hippocampal architecture (data not shown). We first confirmed *Srf* deletion in astrocytes. Co-immunostaining for SRF, S100β, and NeuN revealed robust SRF expression in both the astrocytes and neurons in three- to five-week-old control mice (Fig. 1*A*). In the brain sections of *Srf*^GFAP^CKO mutant mice, there was robust SRF expression in neurons while it was absent in the astrocytes (Fig. 1*A*,*B*). We observed that the antigen retrieval method caused a slightly punctate SRF immunostaining, which was more pronounced in the mutant sections. However, this pattern of staining overlapped with NeuN but not with S100β in the mutant sections (Fig. 1*A*). Quantitative and semi-quantitative real-time PCR using total RNA isolated from whole brain showed decreased *Srf* expression in mutants relative to controls (Fig. 1*C*,*D*).

**Figure 2.**
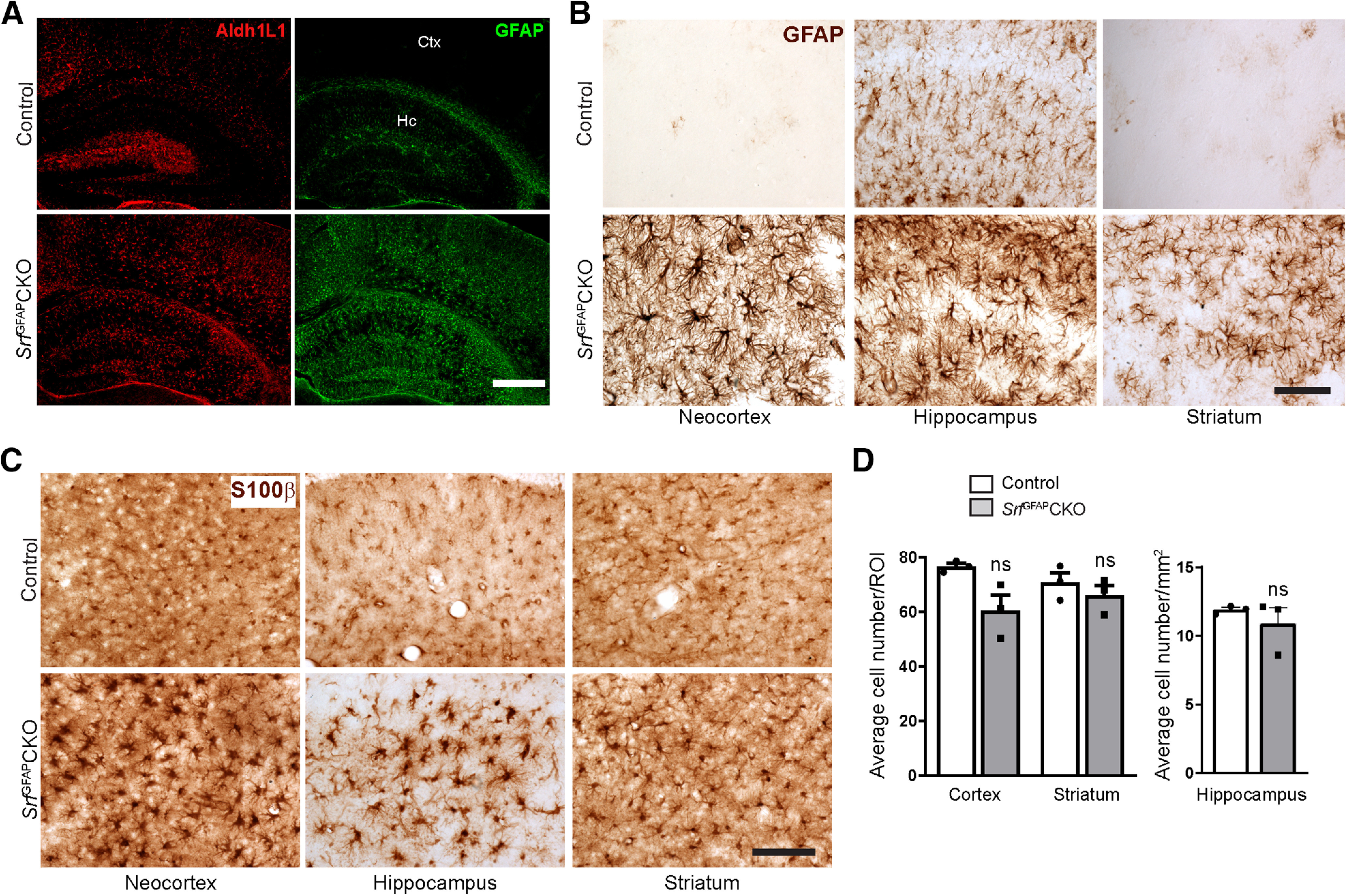
Conditional deletion of SRF in astrocytes results in reactive gliosis. ***A***, Representative images of immunostaining for GFAP and ALDH1L1 in three-week-old *Srf*^GFAP^CKO and control littermates shows higher expression of these markers in neocortex (Ctx) and hippocampus (Hc) of mutant mice as compared with control littermates. ***B***, ***C***, GFAP and S100β immunostaining also showed that the astrocytes in mutant mice were hypertrophic compared with control mice (*n* = 3 mice). ***D***, Quantification of S100β^+^ astrocytes from (***C***). Cortex, control (76.58 ± 1.19), *Srf*^GFAP^CKO (50.25 ± 5.67); striatum, control (70.71 ± 3.61), *Srf*^GFAP^CKO (66.04 ± 3.72); hippocampus, control (11.90 ± 0.17), *Srf*^GFAP^CKO (10.88 ± 1.14; *n* = 3 mice). Scale bars: 100 μm (***A***) and 50 μm (***B***, ***C***); ns, not significant. Two tailed *t* test. Data are mean ± SEM.

We then asked whether *Srf* deletion had any effect on astrocyte development. In three- to five-week-old control mice, astrocytes in hippocampus and fibrous astrocytes in the white matter expressed GFAP, while there was no detectable GFAP expression in the neocortical astrocytes, which have been shown to downregulate GFAP expression postnatally (Buffo et al., 2008; Robel et al., 2009; Fig. 2*A*,*B*). However, the astrocytes in control mice expressed other astrocytic markers, such as S100β (Fig. 2*C*). In striking contrast, astrocytes in the *Srf*^GFAP^CKO mice exhibited pronounced GFAP expression (Fig. 2*A*,*B*) along with hypertrophic morphology as seen from immunostaining for GFAP, Aldh1L1, and S100β (Fig. 2*A–C*), both hallmarks of reactive astrogliosis. Cell counts of S100β^+^ cells revealed no change in the number of astrocytes between control and mutant mice in all regions analyzed (Fig. 2*D*). We had shown earlier that neuron-specific deletion of SRF does not affect astrocyte differentiation or cause reactive gliosis (Lu and Ramanan, 2012). Therefore, although unlikely, any transient Cre expression in neurons by the hGFAP-Cre transgene is unlikely to cause the gliosis phenotype seen in *Srf*^GFAP^CKO mice.

**Figure 3.**
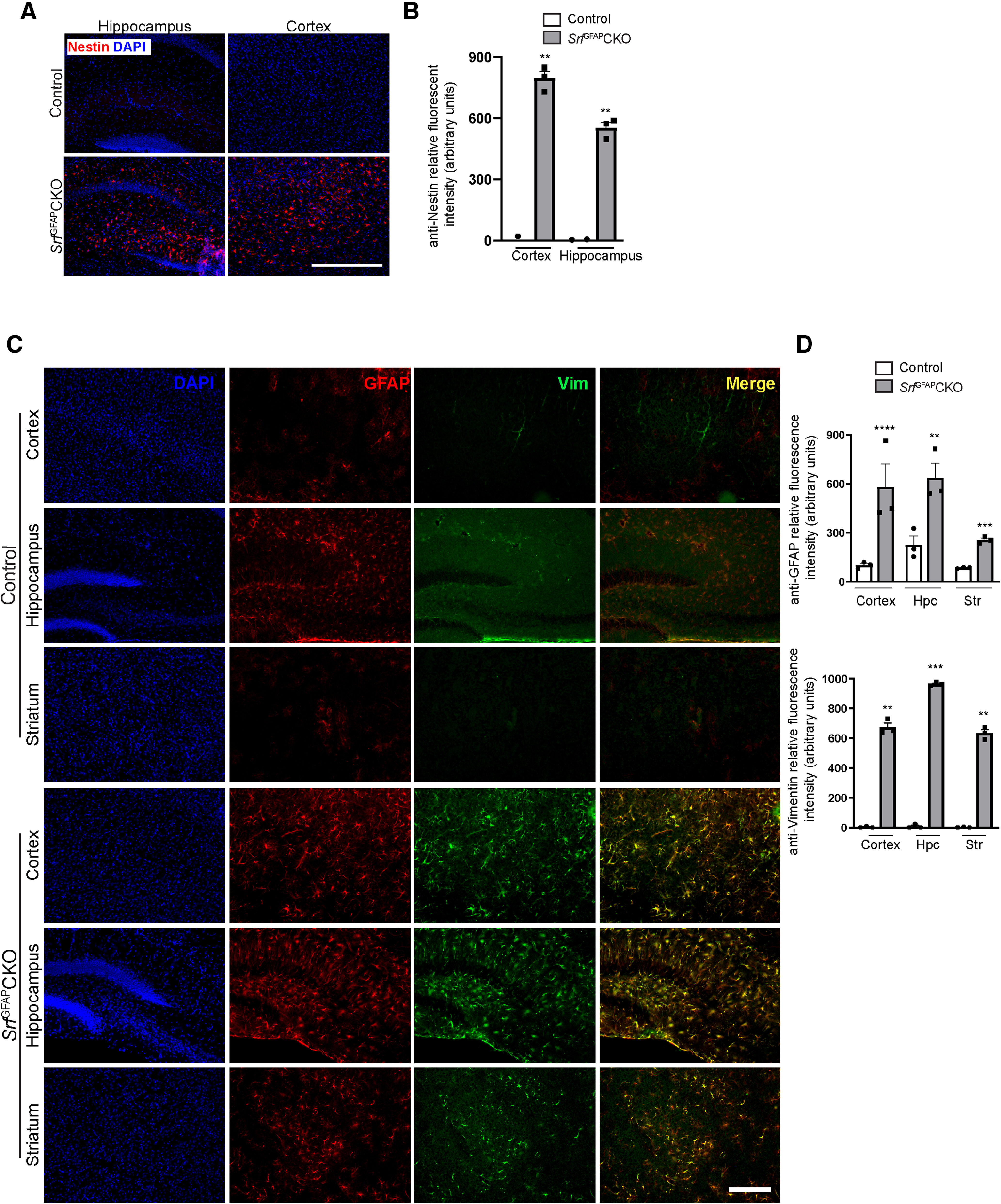
SRF ablation in astrocytes leads to widespread astrogliosis. ***A***, Representative images of immunostaining for the astrogliosis marker, nestin in three-week-old *Srf*^GFAP^CKO and control littermates shows reactive astrocytes in cortex and hippocampus of *Srf* mutant mice but not in control littermates. ***B***, Quantification of nestin fluorescence intensity shown in ***A***. Cortex: control (0.0 ± 0.14), *Srf*^GFAP^CKO (771.9 ± 16.14), hippocampus: control (0.0 ± 8.15), *Srf*^GFAP^CKO (556.1 ± 38.56; *n* = 3 mice). ***C***, Representative images showing co-immunostaining with GFAP and the gliosis marker, vimentin. There is little or no GFAP and vimentin expression in three-week control mice. In contrast, the astrocytes in mutant mice exhibit strong expression and colocalization of GFAP and vimentin. ***D***, Quantification of fluorescence intensity in ***C***. For GFAP, cortex, control (102.1 ± 5.28), *Srf*^GFAP^CKO (580.1 ± 62.10); hippocampus, control (228.0 ± 34.20), *Srf*^GFAP^CKO (638.1 ± 73.97); striatum, control (85.78 ± 1.83), *Srf*^GFAP^CKO (256.9 ± 7.73). For vimentin, cortex, control (2.93 ± 0.99), *Srf*^GFAP^CKO (674.10 ± 27.70); hippocampus, control (12.02 ± 12.02), *Srf*^GFAP^CKO (964.10 ± 11.79); striatum, control (2.33 ± 0.33), *Srf*^GFAP^CKO (633.50 ± 41.37). Shown here are neocortex, hippocampus (Hpc), and striatum (Str; *n* = 3 mice). Scale bars: 500 μm (***A***) and 50 μm (***C***); **p < 0.005, ***p < 0.0005, ****p < 0.0001. Two tailed *t* test. Data are mean ± SEM.

Astrocytes exhibit regional heterogeneity and previous gene deletion studies have shown region-specific generation of reactive astrocytes (Garcia et al., 2010; Kang et al., 2014). We therefore asked whether reactive astrocytes in *Srf*^GFAP^CKO mice were also regionally restricted. For this, we analyzed serial sections from the entire brain of control and *Srf* knock-out mice. We found that astrocytes in most regions of the brain in control mice did not express or weakly expressed GFAP (Fig. 2*A*,*B*). In striking contrast, brain sections from *Srf*^GFAP^CKO mice exhibited intense GFAP expression in all brain regions analyzed including in striatum and corpus callosum (Fig. 2*A*,*B*). To further confirm that the astrocytes in *Srf*^GFAP^CKO mice are indeed reactive, we immunostained for known astrogliosis markers, vimentin and nestin (Ridet et al., 1997). In contrast to control littermates, the *Srf*^GFAP^CKO mice exhibited robust nestin-positive (Fig. 3*A*,*B*) and vimentin-positive (Fig. 3*C*,*D*) astrocytes, thus confirming their reactive state. These observations suggest that astrocyte-specific deletion of *Srf* results in reactive astrocytes.

### Astrogliosis seen in *Srf* knock-out mice is not induced by cell death

**Figure 4.**
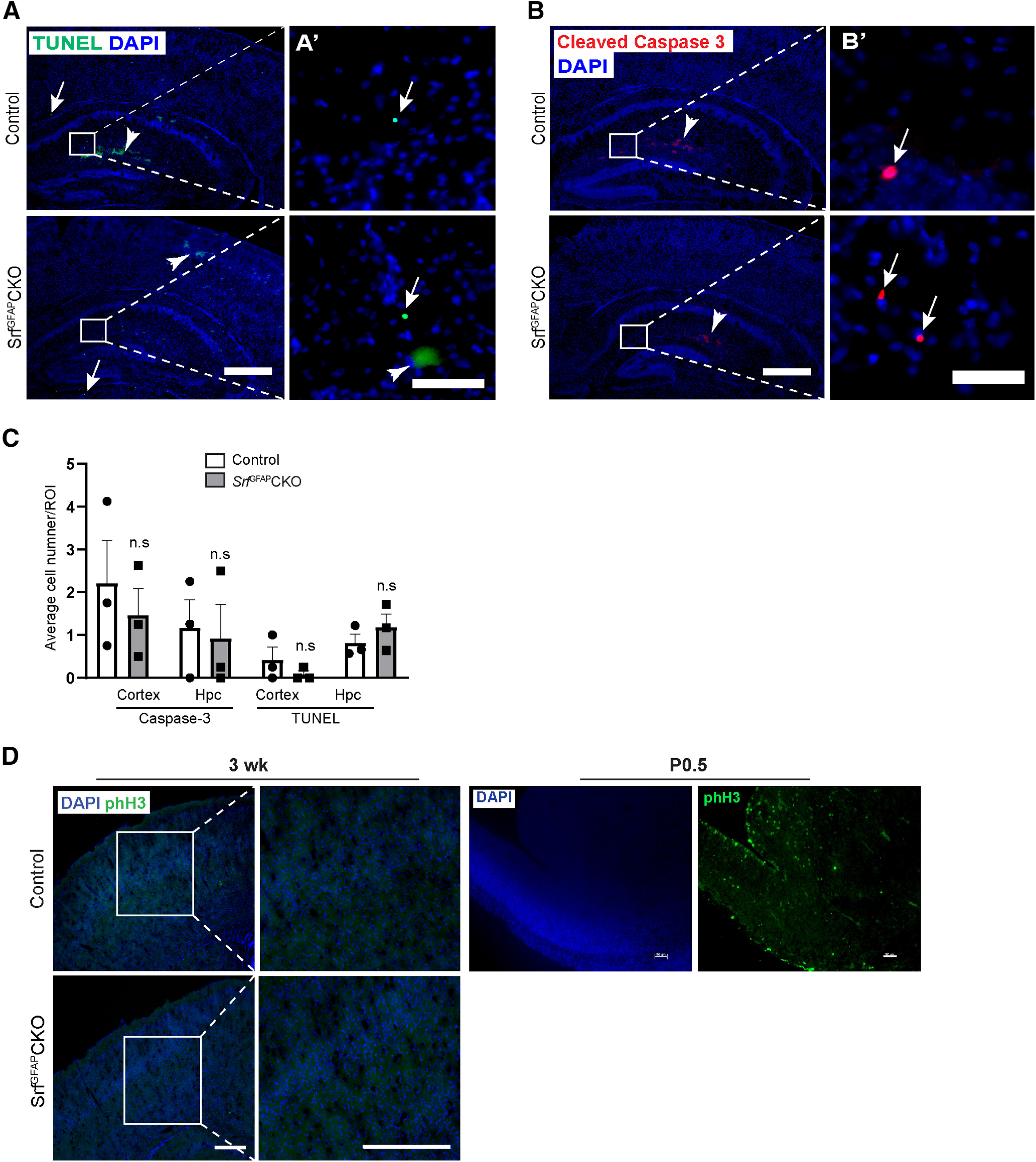
Absence of cell death in *Srf* mutant mice. ***A***, Representative images of TUNEL staining of two-week-old *Srf*^GFAP^CKO mice and control littermates. Amplified view of boxed region is shown on right. Arrows show TUNEL^+^ cells while the arrowhead shows non-specific staining. ***B***, Representative images of immunostaining for cleaved caspase-3 in the neocortex and hippocampus of two-week-old *Srf*^GFAP^CKO mice and control littermates. Amplified view of boxed region is shown on right. Arrows show cleaved caspase 3^+^ cells while the arrowhead shows non-specific staining. ***C***, Quantification of TUNEL^+^ and cleaved caspase-3^+^ cells in neocortex and hippocampus (Hpc) shows the no significant difference in the number of dead cells between *Srf*^GFAP^CKO mice and control littermates. Caspase-3: cortex, control (2.20 ± 1.00), *Srf*^GFAP^CKO (1.45 ± 0.77); hippocampus, control (1.16 ± 0.65), *Srf*^GFAP^CKO (0.91 ± 0.79). TUNEL: cortex, control (0.37 ± 0.21), *Srf*^GFAP^CKO (0.08 ± 0.052); hippocampus, control (0.81 ± 0.21), *Srf*^GFAP^CKO (1.18 ± 0.25; *n* = 3 mice). ***D***, Representative images of immunostaining for the proliferation marker, phH3 in three-week-old *Srf*^GFAP^CKO and their respective control mice showed no proliferating cells in the mutant mice. Immunostaining of P0.5 mouse brain section showed many phH3-positive cells and served as a control; n.s., not significant. Two-tailed *t* test. Data are mean ± SEM. Scale bars: 20 μm (***A’***, ***B’***) and 200 μm (***A***, ***B***) and 100 μm (***D***); *n* = 3 mice.

Astrogliosis is generally induced by several extrinsic factors, such as cell death or a leaky BBB (Pekny and Nilsson, 2005). We first analyzed cell death by immunostaining for cleaved caspase-3 as well as by TUNEL staining. We did not observe any discernible cell death at two weeks of age in *Srf*^GFAP^CKO mice, just before the onset of astrogliosis (Fig. 4*A–C*). This strongly suggested that reactive astrogliosis seen in the *Srf* knock-out mice is not triggered by cell death. During conditions of severe neural injury or trauma, reactive astrocyte undergo proliferation (Sofroniew, 2014). To determine whether SRF-deficient reactive astrocytes are proliferating, brain sections from *Srf*^GFAP^CKO mice and control littermates were immunostained with the proliferation marker, phH3. There were no phH3-positive cells observed in either *Srf* mutant mice or their control littermates, excluding the presence of proliferating astrocytes (Fig. 4*D*).

### BBB is unaffected in *Srf* knock-out mice

**Figure 5.**
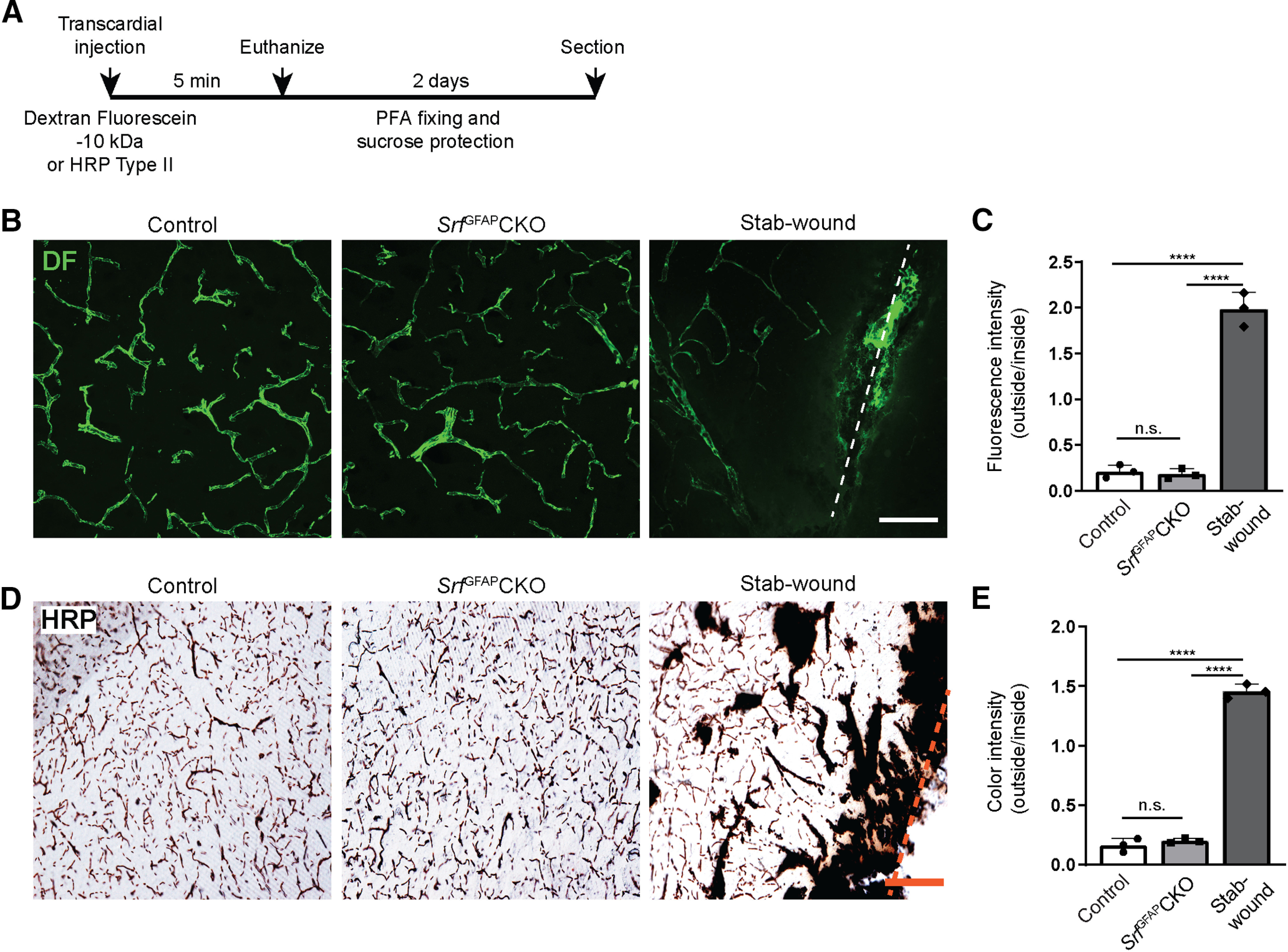
BBB is not compromised in *Srf*^GFAP^CKO mice. ***A***, Schematic diagram showing experimental timeline of dextran fluorescein (DF) and HRP injection and tissue processing. ***B***, 10-kDa TMR-dextran fluorescein (DF) tracer injection reveals normal architecture of cerebral vasculature in three- to five-week-old *Srf*^GFAP^CKO and their control littermates. ***C***, Quantification of ratio of fluorescence intensity outside versus inside the blood vessel reveals no significant difference between *Srf*^GFAP^CKO mice and their control littermates, indicative of intact BBB. A stab-wounded brain served as a control to show BBB leakage. Control (0.21 ± 0.0), *Srf*^GFAP^CKO (0.18 ± 0.05), stab-wound (1.97 ± 0.26; *n* = 3 mice). ***D***, Transcardial injection of 44-kDa HRP Type II in *Srf*^GFAP^CKO mice and their respective controls shows that HRP was restricted to the blood vessel lumen. ***E***, Quantification of ratio of color intensity outside versus inside the blood vessel shows no significant difference between control and mutant mice (*n* = 3 mice). Control (0.16 ± 0.05), *Srf*^GFAP^CKO (0.20 ± 0.02), stab-wound (1.45 ± 0.08). Scale bars: 400 μm (***B***) and 200 μm (***D***); ns, not significant; ****p < 0.0001, one-way ANOVA, data are mean ± SD.

Given the widespread and persistent reactive astrogliosis seen in *Srf* mutant mice, it is possible that a leaky BBB could be the likely cause for the astrogliosis observed in *Srf* knock-out mice (Pekny and Nilsson, 2005). To study BBB integrity, we injected two different tracers, 10-kDa dextran fluorescein (Fig. 5*A–C*) and 44-kDa HRP Type II (Fig. 5*A*,*D*,*E*), into three- to five-week-old *Srf*^GFAP^CKO mutant and control mice (Andreone et al., 2017). We did not observe any discernible presence of these tracers in the brain parenchyma of *Srf* knock-out mice relative to their littermate controls (Fig. 5), supporting an intact BBB.

### Persistent reactive astrogliosis seen in *Srf*^GFAP^CKO mice throughout adulthood

**Figure 6.**
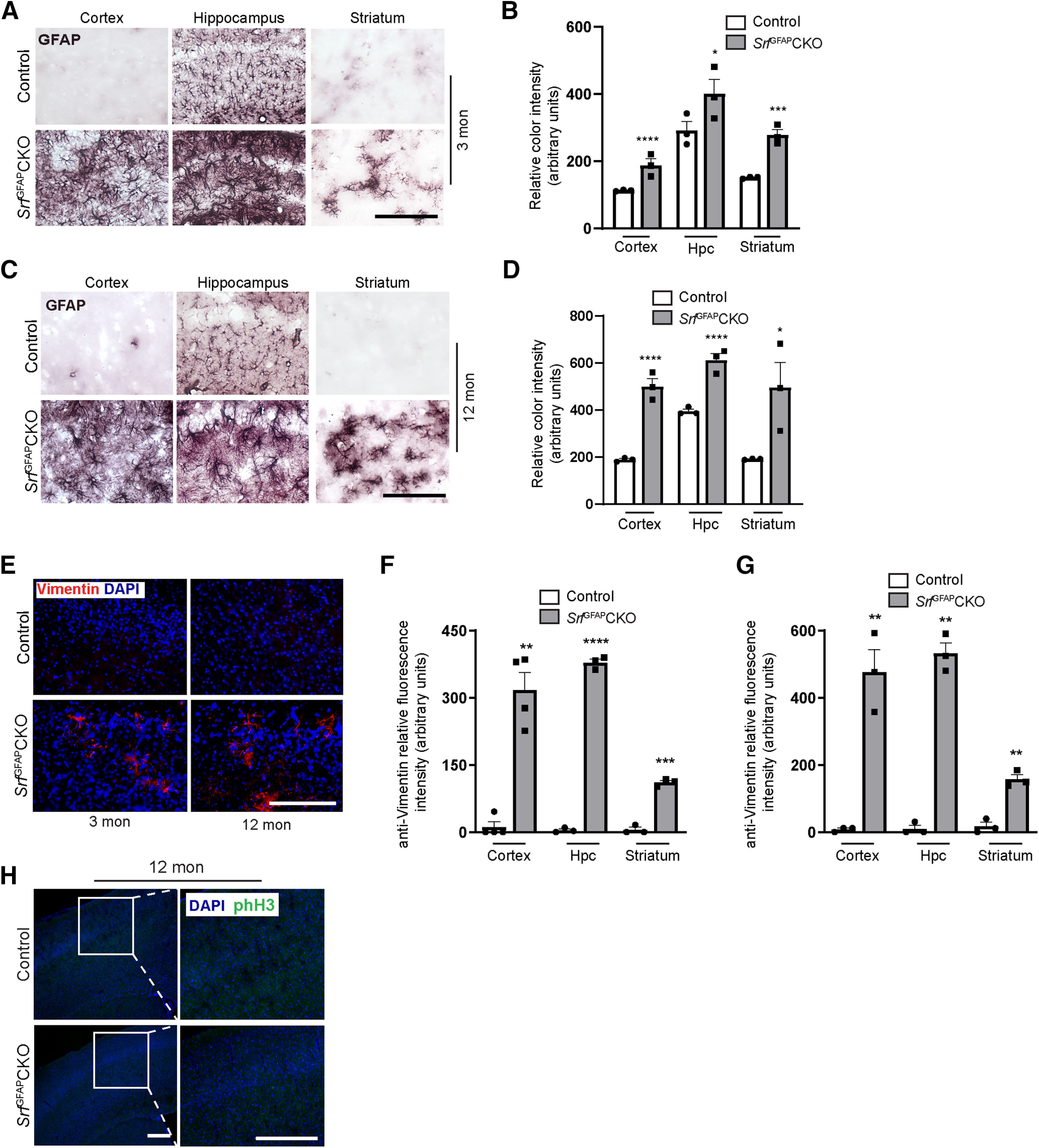
Persistent astrogliosis in *Srf*^GFAP^CKO mice. ***A***, Immunostaining for GFAP in three-month-old, (3-mon) *Srf*^GFAP^CKO mice and control littermates shows widespread astrogliosis in all the brain regions. ***B***, Quantification of color intensity in ***A***. Cortex, control (107.2 ± 0.61), *Srf*^GFAP^CKO (176.4 ± 7.35); hippocampus, (Hpc), control (270.2 ± 9.11), *Srf*^GFAP^CKO (385.4 ± 16.14); striatum, control (151.4 ± 1.280), *Srf*^GFAP^CKO (260.6 ± 18.58; *n* = 3 mice). ***C***, Representative images of immunostaining for GFAP in 12-month-old, (12-mon) *Srf*^GFAP^CKO mice and control littermates shows persistent astrogliosis in all the brain regions analyzed. Shown here are cortex, hippocampus (Hpc), and striatum. ***D***, Quantification of relative color intensity in ***C***. Quantification of color intensity in ***C***; cortex, control (188.2 ± 2.85), *Srf*^GFAP^CKO (536.0 ± 26.03); hippocampus, control (356.3 ± 6.89), *Srf*^GFAP^CKO (591.0 ± 29.43); striatum: control (191.1 ± 4.83), *Srf*^GFAP^CKO (495.7 ± 80.51; *n* = 3 mice). ***E***, Representative images of immunostaining for reactive astrogliosis marker, vimentin, in three- and 12-month-old *Srf*^GFAP^CKO mice and control littermates shows gliosis astrocytes only in the brains of *Srf* mutant mice. ***F***, ***G***, Relative fluorescent intensity of vimentin immunostaining in three-month-old (***F***) and 12-month-old (***G***) mice compared with control littermates. ***F***, Cortex, control (11.52 ± 11.52), *Srf*^GFAP^CKO (317.1 ± 39.27); hippocampus: control (4.47 ± 3.11), *Srf*^GFAP^CKO (378.7 ± 8.17), striatum: control (5.43 ± 5.43), *Srf*^GFAP^CKO (110.8 ± 4.94). ***G***, Cortex, control (8.22 ± 4.11), *Srf*^GFAP^CKO (475.7 ± 67.75); hippocampus, control (10.26 ± 10.26), *Srf*^GFAP^CKO (531.6 ± 31.96); striatum: control (17.30 ± 11.98), *Srf*^GFAP^CKO (157.4 ± 13.96; *n* = 3–4 mice). ***H***, Immunostaining for the proliferation marker, phH3, in 12-month-old *Srf*^GFAP^CKO and control littermates showed no proliferating cells even at this age. Scale bar: 200 μm; *p < 0.05, **p < 0.005, ***p < 0.0005, ****p < 0.0001. Two tailed *t* test. Data are mean ± SEM.

Astrogliosis could be a transient phenomenon, lasting a few days to several weeks, or a long-lasting event resulting in a glial scar, depending on the severity of trauma or injury (Sofroniew, 2014). We therefore asked whether the astrogliosis seen in *Srf*^GFAP^CKO mice is a transient process. To address this, we immunostained brain sections from three- and 12-month-old control and knock-out mice. The astrocytes in hippocampus of control mice showed normal GFAP expression while there was very faint or no GFAP expression in the astrocytes in the other brain regions both at either three (Fig. 6*A*,*B*) or 12 (Fig. 6*C*,*D*) months of age. In contrast, the astrocytes in the knock-out mice, at both three and 12 months of age, expressed intense GFAP expression and exhibited hypertrophy in all brain regions (Fig. 6*A–D*) similar to that seen at three weeks (Figs. 2, 3). To further confirm astrogliosis, we immunostained for vimentin and found robust expression only in the astrocytes in the knock-out mice but not in their control littermates (Fig. 6*E–G*). Immunostaining for phH3 did not reveal any positive cells, suggesting that these astrocytes are also not proliferative in older mice (Fig. 6*H*). Together, these observations demonstrate that SRF ablation in astrocytes results in widespread astrogliosis that persists through adulthood.

### Microglial activation in *Srf*^GFAP^CKO mice

**Figure 7.**
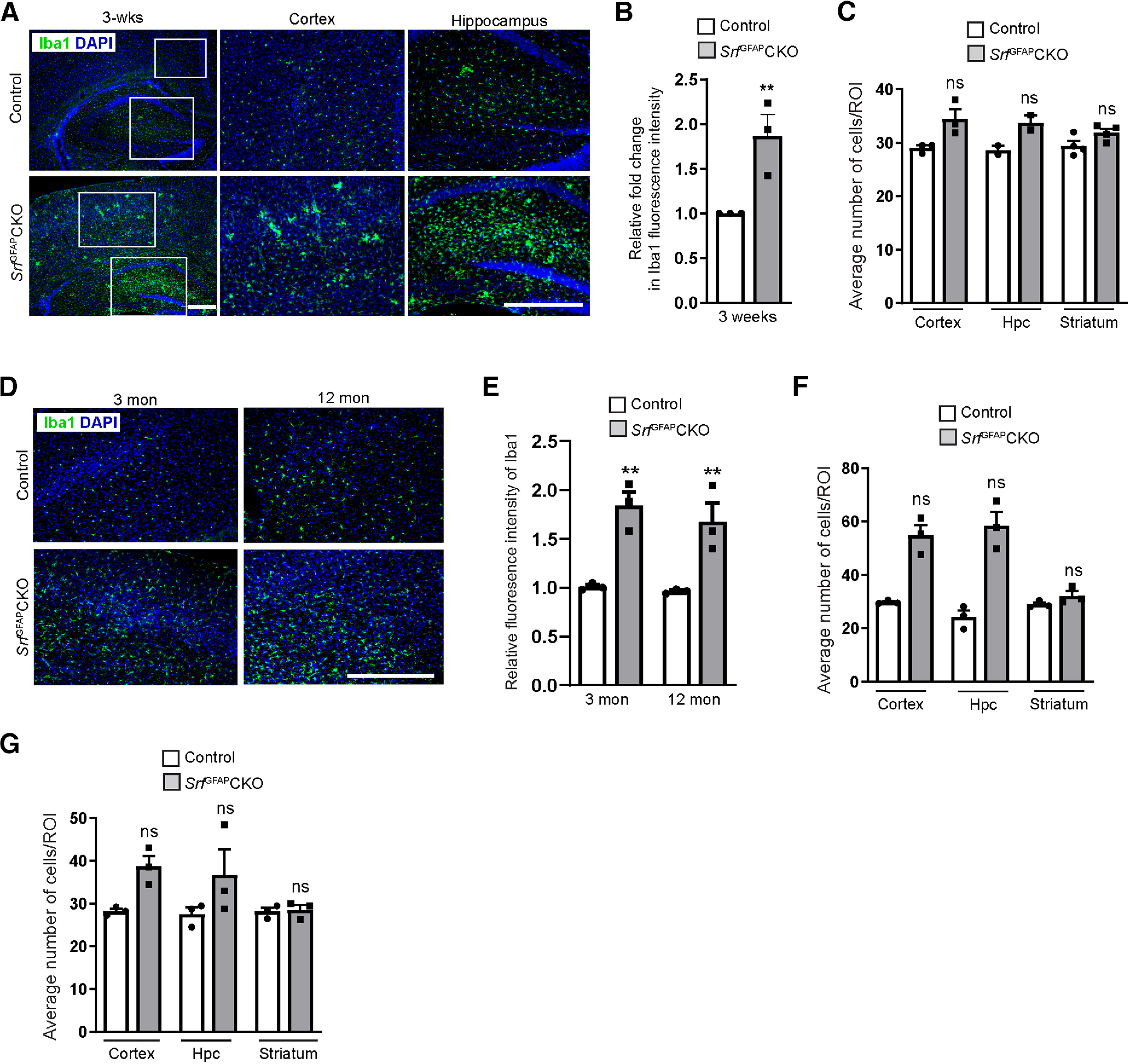
Microglial activation is seen along with astrogliosis in *Srf*^GFAP^CKO mice. ***A***, Immunostaining for the microglia marker, Iba1, showed increased Iba1 expression in the neocortex and hippocampus of mutant mice as compared with the control littermates, suggesting activated microglia in mutant brain. ***B***, Relative fluorescence intensity of Iba1 in the neocortex of *Srf* mutant mice compared with control littermates in ***A*** shows a significant increase in Iba1 expression in the *Srf* mutants indicative of microgliosis. Control (1.0 ± 0.0), *Srf*^GFAP^CKO (1.97 ± 0.19; *n* = 3 mice). ***C***, Quantification of Iba1^+^ cells in three-week-old control and mutant mice. Cortex, control (29.08 ± 0.43), *Srf*^GFAP^CKO (34.4 ± 2.68); hippocampus (Hpc), control (28.64 ± 0.82), *Srf*^GFAP^CKO (33.80 ± 1.40); striatum, control (29.46 ± 1.20), *Srf*^GFAP^CKO (32.0 ± 0.42; *n* = 3 mice). ***D***, Representative images of sections immunostained for microglial marker Iba1 in aged mice (three- and 12-month-old) in *Srf*^GFAP^CKO and control littermates show increased expression of Iba1 in the *Srf* mutants. Shown here is neocortex. ***E***, Relative fluorescence intensity of Iba1 in the neocortex of *Srf* mutant mice compared with control littermates shows persistent microgliosis in the *Srf* mutants throughout adulthood. Three months, control (1.0 ± 0.0), *Srf*^GFAP^CKO (1.8 ± 0.22); 12 months, control (1.0 ± 0.0), *Srf*^GFAP^CKO (1.71 ± 0.17; *n* = 3 mice). ***F***, ***G***, Quantification of Iba1^+^ cells at three months (***F***), cortex, control (30.00 ± 0.62), *Srf*^GFAP^CKO (54.50 ± 6.87); hippocampus (Hpc), control (24.00 ± 4.25), *Srf*^GFAP^CKO (58.75 ± 9.00); striatum, control (29.25 ± 1.25), *Srf*^GFAP^CKO (32.75 ± 3.00); and 12 months (***G***) cortex, control (28.25 ± 0.57), *Srf*^GFAP^CKO (38.75 ± 2.49); hippocampus, (Hpc), control (27.58 ± 1.55), *Srf*^GFAP^CKO (36.75 ± 6.00); striatum, control (28.17 ± 0.88), *Srf*^GFAP^CKO (28.58 ± 1.17; *n* = 3 mice). Scale bars: 200 μm; **p < 0.005; ns, not significant. Two tailed *t* test. Data are mean ± SEM.

Reactive astrocytes are often observed along with microgliosis (Frank and Wolburg, 1996; Zhang et al., 2010). We therefore asked whether microglia exhibited a reactive state in the *Srf* knock-out mice. Immunostaining for the microglial marker, Iba1 showed increased expression in brain sections from three-week-old mutant mice, relative to control littermates, indicative of reactive microglia (Fig. 7*A*,*B*). We then quantified the number of Iba1^+^ cells and found no significant difference in microglial numbers between control and knock-out mice (Fig. 7*C*). However, we noticed that the microglia tend to form clusters in the cortex and hippocampus (Fig. 7*A*). We next asked whether microgliosis also persisted throughout adulthood similar to that seen for astrogliosis. As seen at three weeks, brain sections from three- and 12-month-old *Srf*^GFAP^CKO mice also showed strong upregulation of IbaI expression relative to that from control mice (Fig. 7*D*,*E*), suggesting that microglia also exhibited a reactive state along with reactive astrocytes. Similar to that seen at three weeks of age, we did not find any difference in the number of Iba1^+^ cells in both three- and 12-month-old *Srf*^GFAP^CKO mice compared with control littermates (Fig. 7*F*,*G*). Immunostaining for proliferation marker, phH3 did not reveal any positive cells (Figs. 4*D*, 6*H*), indicating that these reactive microglia are also not proliferative.

### Persistent gliosis in *Srf*^GFAP^CKO mice does not affect neuronal viability

**Figure 8.**
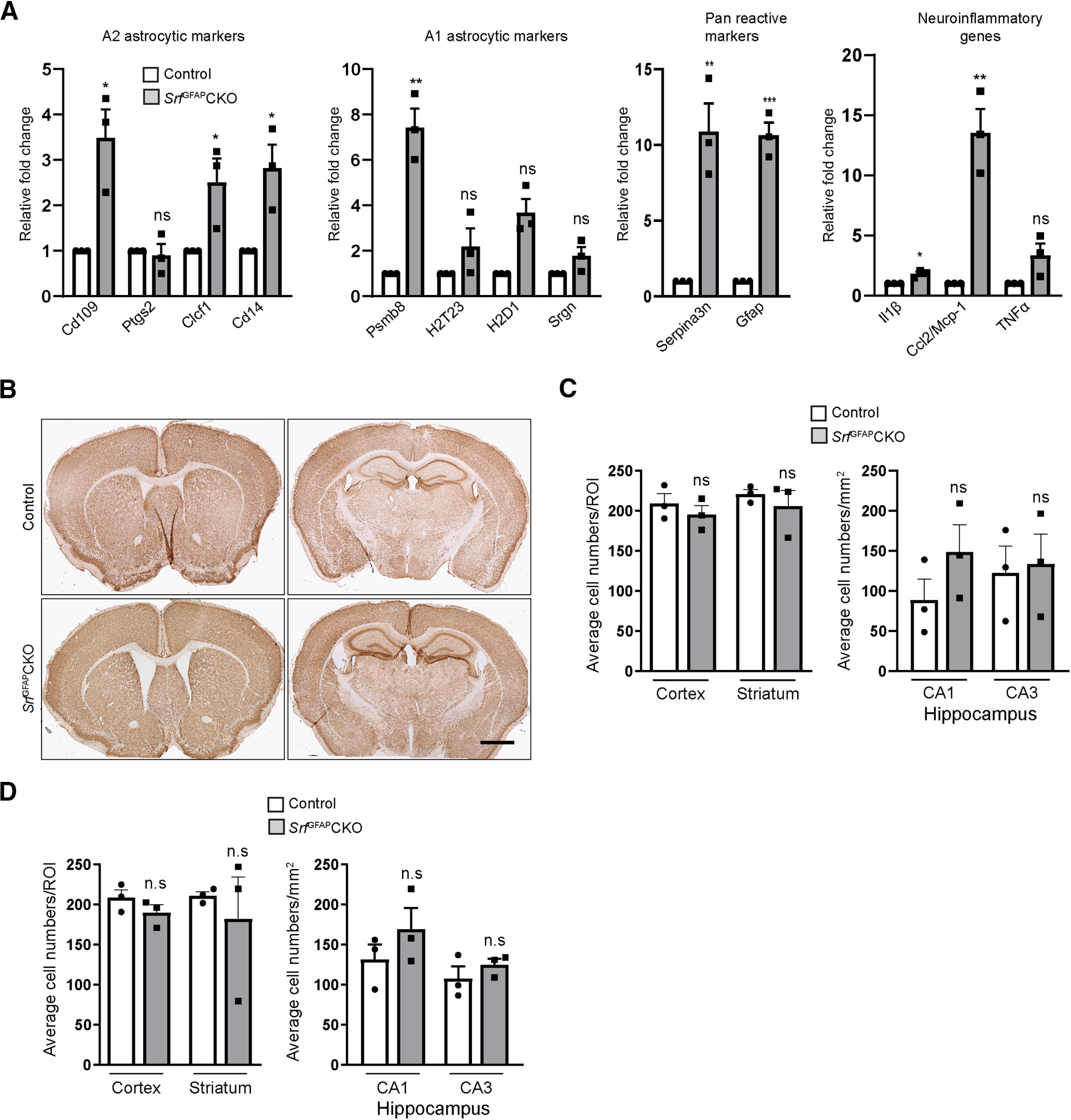
Prolonged gliosis in *Srf*^GFAP^CKO mice does not affect neuronal survival. ***A***, Quantitative real-time PCR for A1, A2, pan-reactive astrocyte markers and neuroinflammatory genes shows expression of a greater number of A2 reactive genes in the brains of *Srf* mutant mice compared with A1 genes. The mutant astrocytes also exhibited a higher expression of neuroinflammatory genes, *Il1β* and *Ccl2/Mcp-1* but not *TNFα*. Control (1.0 ± 0.0), *Srf*^GFAP^CKO, *Cd109* (3.49 ± 0.62), *Clcf1* (2.51 ± 0.52), *Cd14* (2.82 ± 0.51), *Ptgs2* (0.90 ± 0.25), *Psmb8* (7.43 ± 0.83), *H2T23* (2.20 ± 0.78), *H2D1* (2.4 ± 0.65), *Srgn* (1.78 ± 0.39), *Serpina3n* (10.88 ± 1.86), *Gfap* (9.62 ± 0.84), *Il1β* (1.806 ± 0.16), *Ccl2/Mcp-1* (13.52 ± 1.967), *TNFα* (3.35 ± 0.97; *n* = 3 mice). ***B***, Representative images of immunostaining for the neuronal marker, NeuN shows normal structural integrity in three- to five-week-old *Srf*^GFAP^CKO mice compared with control littermates. ***C***, ***D***, Quantification of NeuN^+^ cells in three- to five-week-old (***C***) and 12-month-old (***D***) control and mutant mice shows no significant change in neuronal numbers in the mutant mice. ***C***, Cortex, control (209.5 ± 12.10), *Srf*^GFAP^CKO (195.2 ± 11.18); CA1, control (78.25 ± 16.48), *Srf*^GFAP^CKO (148.4 ± 34.10); CA3, control (124.4 ± 24.47), *Srf*^GFAP^CKO (133.5 ± 37.20); striatum, control (220.8 ± 5.90), *Srf*^GFAP^CKO (205.8 ± 19.59; *n* = 3 mice). ***D***, Cortex, control (208.4 ± 9.87), *Srf*^GFAP^CKO (190.0 ± 9.71); CA1, control (119.0 ± 22.36), *Srf*^GFAP^CKO (148.2 ± 12.23); CA3, control (101.0 ± 22.05), *Srf*^GFAP^CKO (127.8 ± 12.19); striatum, control (211.1 ± 5.14), *Srf*^GFAP^CKO (182.1 ± 51.89; *n* = 3 mice), Scale bar, 1 mm; *p < 0.05, **p < 0.005, ***p < 0.0005; ns, not significant. Two tailed *t* test. Data are mean ± SEM.

Recent studies have shown that reactive astrocytes can be broadly classified as either neurotoxic (A1) or neuroprotective (A2) depending on the external stimuli (Zamanian et al., 2012; Liddelow et al., 2017). In order to determine the phenotypic state of SRF-deficient reactive astrocytes, we performed quantitative RT-PCR for some of the A1 (*Psmb8*, *H2T23*, *H2D1*, *Srgn*), A2 (*Cd109*, *Ptgs2*, *Clcf1*, *Cd14*), and pan-reactive marker genes (*Serpina3n*, *Gfap*). Although we found that the *Srf*^GFAP^CKO mice expressed both A1 and A2 genes, there were more A2 reactive astrocyte marker genes that were upregulated compared with A1 genes suggesting that the SRF-deficient reactive astrocytes are likely to be A2-like (Fig. 8*A*). Since we also observed microgliosis, we next assessed the expression of neuroinflammatory genes. Quantitative PCR showed a significant increase in the expression of *Il1β* and *Ccl2/Mcp-1* but not *TNFα* in the brains of *Srf*^GFAP^CKO mice (Fig. 8*A*), suggesting that these genes could be one of the underlying causes of microgliosis observed in the *Srf* mutant mice.

We next asked whether prolonged gliosis in the SRF-GFAP mutant mice affected neuronal numbers. For this, five-week-old and 12-month-old brain sections from *Srf*^GFAP^CKO mice and control littermates were immunostained for the neuronal marker, NeuN. We did not observe any significant difference in NeuN-positive cells in the brains of mutant and control mice at both these ages (Fig. 8*B–D*). This suggested that persistent gliosis observed in the brains of *Srf*^GFAP^CKO mice did not affect neuronal survival and that SRF-deficient reactive astrocytes are likely not neurotoxic.

## Discussion

Reactive astrogliosis is an important cellular response to neuronal injury, infection and neurodegeneration in the CNS, and this is critical to reduce inflammation, restrict tissue damage and cell death, and promote tissue repair (Sofroniew, 2005; Pekny and Pekna, 2014). Currently, very little is known about the cell-intrinsic mechanisms that regulate the conversion of an astrocyte from a non-reactive to a reactive state. In this study, we show that deletion of the transcription factor, SRF in astrocytes results in widespread reactive gliosis in the brain starting three weeks of age. The reactive astrocytes persisted along with microgliosis throughout adulthood and both astrocytes and microglia did not exhibit proliferation. Our results suggest that SRF is required in a cell-autonomous manner to regulate reactive astrogliosis in the mammalian brain.

SRF is a ubiquitously expressed transcription factor that has been shown to play critical roles in several aspects of nervous system development and function (Knöll and Nordheim, 2009). Deletion of SRF in developing and adult neurons resulted in deficits in neuronal migration, axon growth, hippocampal circuit formation, activity-dependent gene expression, and learning and memory (Alberti et al., 2005; Ramanan et al., 2005; Etkin et al., 2006; Knöll et al., 2006; Wickramasinghe et al., 2008; Stritt and Knöll, 2010; Johnson et al., 2011; Lu and Ramanan, 2011; Li et al., 2014). Deletion of SRF within neural stem cells specifically affected differentiation to both astrocytes and oligodendrocytes (Lu and Ramanan, 2012). Interestingly, neuronal SRF deletion did not affect astrocyte differentiation or cause any reactive astrogliosis (Lu and Ramanan, 2012) but revealed a paracrine effect of neuronal SRF on oligodendrocyte maturation and myelination (Stritt et al., 2009; Anastasiadou et al., 2015). Currently, the role of SRF in astrocyte development remains poorly understood. This study identifies a critical role for SRF in maintenance of astrocytes in a non-reactive state. However, it is possible that since SRF deletion in the *Srf*^GFAP^CKO mice likely starts around E16.5 during embryonic development, the reactive astrogliosis could be because of developmental deficits.

Previous studies have shown that genetic ablation of the extracellular matrix protein, β1-integrin (Itgβ1) in astrocytes results in astrogliosis starting four weeks of age and Itgβ1 mutant mice exhibit spontaneous seizures (Robel et al., 2009, 2015). Attenuation of sonic hedgehog (Shh) signaling in postnatal astrocytes resulted in reactive astrocytes that were restricted to the forebrain alone suggesting a role for Shh in maintaining the non-reactive state of specific astrocytic populations (Garcia et al., 2010). Similarly, attenuation of fibroblast growth factor (FGF) signaling by deletion of FGF receptors, FGFR1–3, resulted in astrogliosis that was restricted to the neocortex and hippocampus although deletion occurred in other regions as well (Kang et al., 2014). Interestingly, expression of a dominant negative FGFR3 (dnFGFR3) and a constitutively active FGFR3 (caFGFR3) in astrocytes produced different results. While caFGFR3 suppressed astrogliosis in one study, it resulted in enlarged astrocytes with increased branching in another (K. Kang et al., 2014; W. Kang et al., 2014). Furthermore, expression of a dnFGFR3 suppressed GFAP expression and hypertrophic morphology (K. Kang et al., 2014). Nevertheless, these observations suggest an important role for FGF signaling in regulating the reactive state of forebrain astrocytes. Since reactive astrocytes can either be beneficial or detrimental to normal functioning of the nervous system, this process is expected to be tightly regulated to maintain astrocytes in a non-reactive state.

Reactive astrocytes are induced or regulated by several extracellular signals including cytokines, growth factors, endothelin, purines, and lipopolysaccharide (LPS), and these factors are released by neural and non-neural cells in the CNS following neuronal injury or in response to infection (Kang and Hébert, 2011; Sofroniew, 2014). In the *Srf*^GFAP^CKO mice, we did not observe any cell death when analyzed at two weeks of age (Fig. 4*A–C*); and therefore, cell death is unlikely to be the cause of gliosis, which was first observed starting at three weeks of age. Furthermore, there were no discernible deficits observed in BBB permeability indicating that reactive astrocytes in the *Srf* mutant mice are not induced by a leaky BBB (Fig. 5).

Depending on the severity of neural injury, astrogliosis can manifest as mild, moderate, or severe without or with scar formation (Pekny and Pekna, 2014; Sofroniew, 2014). During mild and moderate astrogliosis, astrocytes exhibit hypertrophy, do not proliferate and return to non-reactive state once the underlying cause is resolved (Pekny and Pekna, 2014; Sofroniew, 2014). In severe astrogliosis, astrocytes exhibit proliferation and can also result in scar formation with significant tissue rearrangement. In the *Srf*^GFAP^CKO, the astrocytes exhibited hypertrophy but were not proliferative when assessed in young and old mice (Figs. 4*D*, 6*H*). The absence of proliferative hypertrophic reactive astrocytes has been reported in mutant mice carrying astrocyte-specific deletion of genes such as Shh (Garcia et al., 2010); β1-integrin (Robel et al., 2009) and FGFR (Kang et al., 2014). Furthermore, the astrogliosis in these mutant mice was found to be restricted to specific regions in the brain likely reflective of their requirement in specific astrocyte populations. In contrast, in the *Srf* knock-out mice, we observed widespread astrogliosis in all regions of the brain including white matter astrocytes (Fig. 6*A–G*). This suggests that conditional *Srf* ablation in astrocytes results in conversion to reactive astrocytes regardless of their regional heterogeniety (Zhang and Barres, 2010).

The *Srf* mutant mice also exhibited reactive microglia starting around three weeks of age and persisted throughout adulthood (Fig. 7). Reactive astrogliosis has been shown to accompany reactive microglia and vice versa (Frank and Wolburg, 1996; Zhang et al., 2010). Reactive astrocytes are known to secrete cytokines, which can cause the activation of microglia (Davalos et al., 2005). Since there appears to be no injury or infection in *Srf*^GFAP^CKO mice, the microglial activation is very likely to be caused by the reactive astrocytes.

Studies have shown that depending on the kind of neuronal injury or insult, reactive astrocytes can be broadly classified as neurotoxic (A1) or neuroprotective (A2; Li et al., 2008; Zamanian et al., 2012; Liddelow et al., 2017). Since the SRF-deficient astrocytes exhibited hypertrophy and enhanced GFAP and vimentin expression (Figs. 2, 3), we looked at the expression of A1 and A2 astrocyte markers. We found expression of more A2 marker genes as compared with A1 genes in the brains *Srf*^GFAP^CKO mice suggesting that SRF-deficient reactive astrocytes are likely to be A2-type (Fig. 8*A*). A1 reactive astrocytes have been shown to secrete neurotoxic factor(s) that resulted in neuronal cell death in cultured neurons (Liddelow et al., 2017). The presence of reactive microglia suggested that the reactive astrocytes could induce microgliosis. We found an increased expression of *Il1β* and *Ccl2/Mcp-1* but not *TNFα* in brains of *Srf*^GFAP^CKO mice (Fig. 8*A*). IL-1β and the chemokine, CCL2/MCP-1 have been shown to induce reactive microglia and could be the underlying cause for prolonged microgliosis (Selenica et al., 2013; Liu and Quan, 2018). Since the *Srf*^GFAP^CKO mice exhibited prolonged gliosis, we analyzed for neuronal numbers and found no significant change in the number of neurons indicating that the reactive astrocytes in the brains of these *Srf* mutant mice do not affect neuronal survival (Fig. 8*A–D*).

In summary, we have identified SRF as a critical regulator of reactive astrocytes in the mouse brain. Ablation of *Srf* in astrocyte-specific manner results in persistent and widespread astrogliosis. Since astrocytes in all brain regions become reactive, it strongly suggests that SRF is important for maintaining both white matter and gray matter astrocytes in a non-reactive state. The SRF-deficient reactive astrocytes appear to be A2-like and the brains of *Srf*^GFAP^CKO mice exhibited normal neuronal numbers despite persistent gliosis. It is reasonable to speculate that SRF expression needs to be downregulated for an astrocyte to become reactive. It is also possible that SRF-dependent transcription is likely affected in reactive astrocytes observed in neurodegenerative disorders. Future studies aimed at identification of SRF target genes may provide novel insights into the mechanisms regulating reactive astrogliosis and may provide potential targets for astrocyte-targeted therapeutics.

## Synthesis

Reviewing Editor: Cheryl Sisk, Michigan State University Decisions are customarily a result of the Reviewing Editor and the peer reviewers coming together and discussing their recommendations until a consensus is reached. When revisions are invited, a fact-based synthesis statement explaining their decision and outlining what is needed to prepare a revision will be listed below. The following reviewer(s) agreed to reveal their identity: Marija Cvetanovic.

Authors have addressed many of the prior concerns and manuscript is much improved. There are several remaining concerns that should be addressed in a revised manuscript.

1. Please include individual data points/values in all histograms in all figures.

2. In Fig 1A neither “the robust SRF expression in astrocytes” nor the loss of SRF in astrocytes in KO mice are easy to see. This is important to show to validate selective SRF knock-out in astrocytes. To help with this could authors please include single color images in addition to merge image (so SRF only, GFAP only and merge)? Or include more representative image?

3. Could authors include DAPI images in their figures to confirm that when they compare intensities/cell counts they are from similar brain regions in wt and KO mice (i.e. for the hippocampus -which part was used to quantify intensity: DG, CA1, CA2)? For instance GFAP intensity in hippocampus (dentate gyrus) in WT mice is pretty good in Figures 1D and 1G, but in Fig 2C there is no GFAP reactivity in hippocampus of WT mice?

4. In Figures 3A and B there are regions of intense TUNEL and caspase 3 staining in WT mice but not in KO mice. However, in histograms there is no difference in cells + for TUNEL or Caspase 3. Were the regions of intense staining used for quantification? If yes, then authors should include more representative images.

5. For consistency, could authors present absolute number of cells positive for Tunel, and caspase 3 per ROI as they did in other figures?

6. Were ROI sizes same for NeuN, S100 and Iba1 counts?

7. Author’s new results in Fig 7 suggest a very interesting concept: that these reactive astrocytes express more neuroprotective A2 genes, which could indicate that SRF negatively regulates neuroprotective gliosis. However, they at the same time also induce microgliosis but neither prolonged astro- nor microgliosis caused any neuronal loss. This is potentially very intriguing finding that warrants looking into more astroglial/microglial genes. We suggest at least examining expression of neuroinflammatory genes such as TNFalpha, IL1B and MCP-1.
